# Primary Breast Extranodal Marginal Zone Lymphoma in Primary Sjögren Syndrome: Case Presentation and Relevant Literature

**DOI:** 10.3390/jcm9123997

**Published:** 2020-12-10

**Authors:** Giuseppe Ingravallo, Eugenio Maiorano, Marco Moschetta, Luisa Limongelli, Mauro Giuseppe Mastropasqua, Gisella Franca Agazzino, Vincenzo De Ruvo, Paola Tarantino, Gianfranco Favia, Saverio Capodiferro

**Affiliations:** 1 Department of Emergency and Organ Transplantation—Section of Pathology, University of Bari Aldo Moro, Piazza G. Cesare, 11, 70124 Bari, Italy; eugenio.maiorano@uniba.it (E.M.); mauro.mastropasqua@uniba.it (M.G.M.); gisella.agazzino@libero.it (G.F.A.); tarpa80@gmail.com (P.T.); 2 Department of Emergency and Organ Transplantation—Breast Unit, University of Bari Aldo Moro, Piazza G. Cesare, 11, 70124 Bari, Italy; marco.moschetta@uniba.it (M.M.); vincenzo.deruvo@yahoo.it (V.D.R.); 3 Department of Interdisciplinary Medicine—Section of Odontostomatology, University of Bari Aldo Moro, Piazza G. Cesare, 11, 70124 Bari, Italy; lululimongelli@gmail.com (L.L.); gianfranco.favia@uniba.it (G.F.); capodiferro.saverio@gmail.com (S.C.)

**Keywords:** autoimmune diseases, Sjögren syndrome, minor salivary glands, B-cell lymphoma, extranodal marginal zone lymphoma, MALT lymphoma, primary breast lymphoma

## Abstract

The association between autoimmune diseases, mostly rheumatoid arthritis, systemic lupus erythematosus, celiac disease and Sjögren syndrome, and lymphoma, has been widely demonstrated by several epidemiologic studies. By a mechanism which has not yet been entirely elucidated, chronic activation/stimulation of the immune system, along with the administration of specific treatments, may lead to the onset of different types of lymphoma in such patients. Specifically, patients affected by Sjögren syndrome may develop lymphomas many years after the original diagnosis. Several epidemiologic, hematologic, and histological features may anticipate the progression from Sjögren syndrome into lymphoma but, to the best of our knowledge, a definite pathogenetic mechanism for such progression is still missing. In fact, while the association between Sjögren syndrome and non-Hodgkin lymphoma, mostly extranodal marginal zone lymphomas and, less often, diffuse large B-cell, is well established, many other variables, such as time of onset, gender predilection, sites of occurrence, subtype of lymphoma, and predictive factors, still remain unclear. We report on a rare case of primary breast lymphoma occurring three years after the diagnosis of Sjögren syndrome in a 57-year-old patient. The diagnostic work-up, including radiograms, core needle biopsy, and histological examination, is discussed, along with emerging data from the recent literature, thus highlighting the usefulness of breast surveillance in Sjögren syndrome patients.

## 1. Introduction

Sjögren’s syndrome (SS) is the second most common autoimmune disease; it is usually classified as primary or secondary to rheumatoid arthritis and other autoimmune diseases, such as lupus erythematosus, sclerodermia, vasculitis, etc., mainly involves the exocrine glands (salivary and lacrimal glands) and is characterized by progressive infiltration of T-and B-lymphocytes [1,2]. The common detectability of hyper-gamma-globulinemia and different autoantibodies (such as rheumatoid factor, anti-Sjögren’s syndrome A and B antibodies) in the blood of SS patients underlines the relevance of B-cell hyperactivity in the pathogenesis [2,3]. Common clinical findings in SS patients are kerato-conjunctivitis sicca, xerostomia, angular cheilitis, and additional symptoms related to the qualitative/quantitative reduction of exocrine secretions [3]. Along with dryness, SS patients may show disabling symptoms, such as fatigue and pain, but also develop systemic manifestations in up to 30–50% of cases, including renal, lung, or neurological disorders [4,5]. In addition, SS patients have an increased risk of lymphoma, such as marginal zone lymphoma (MZL) or mucosal-associated lymphoid tissue (MALT) lymphoma [6,7,8,9,10]. The World Health Organization in 2016 classified MZLs into three distinct types, according to the involved sites: extranodal MZL of MALT (generally termed as MALT lymphoma), nodal MZL, and splenic MZL [11].

The worldwide incidence of SS is difficult to assess as many cases remain undiagnosed for years [12,13]. Overall, extranodal MALT lymphomas more frequently affect the stomach, spleen, thyroid, ocular adnexal tissues, and salivary glands, while they are rare in the breast (1.7–2.2% of primary breast lymphomas), possibly due to the anecdotic presence of MALT tissue at this site [14,15].

Moreover, SS patients may be affected by non-Hodgkin lymphomas (NHL) over the course of the disease; less than 20% are diffuse B-cell lymphomas while the most frequent are of the MALT type (up to 60%), the latter more commonly involving the minor and major salivary glands, pharynx, stomach, small intestine, and thyroid, with an incidence 10–44 times higher than in the general population [4,5,8,9,10,16,17].

We report on a case of an extranodal marginal zone lymphoma of MALT, occurring in the breast of a Caucasian woman, with a three-year history of Sjögren’s syndrome; also, data from the literature on this topic have been collected and reviewed.

## 2. Case Presentation

**Figure 1:**
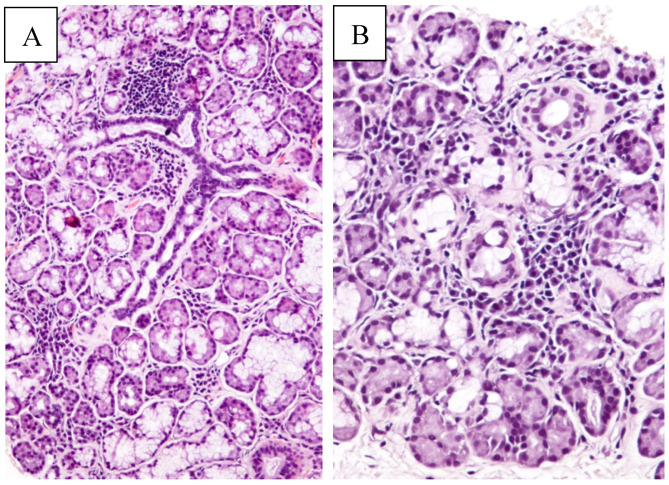
Low power magnification of minor salivary gland biopsy ((**A**): hematoxylin and eosin, original magnification 100×); at higher magnification, small lymphocyte and plasma cell aggregates (i.e., more than one lymphocytic focus) associated with mildly collagenized stroma are detectable ((**B**): hematoxylin and eosin, original magnification 200×).

A 57-year-old Caucasian female was referred to the breast care unit of the Policlinic Hospital of the University of Bari Aldo Moro for a small mass in her right breast. The patient had been suffering from persistent and severe dry eyes and moderate dry mouth for several years. Three years earlier, a biopsy of the minor salivary glands, along with the presence of anti-Sjögren’s syndrome A and B (anti-SSA/SSB) antibodies, lead to the diagnosis of primary SS, in the absence of other autoimmune diseases, as detected by clinical examination and serological tests. The revision of the original histopathological preparations confirmed the diagnosis of lymphocytic sialadenitis with a focus score >1/4 mm^2^, grade 4 according to Chisholm and Mason (Figure 1A,B).

**Figure 2:**
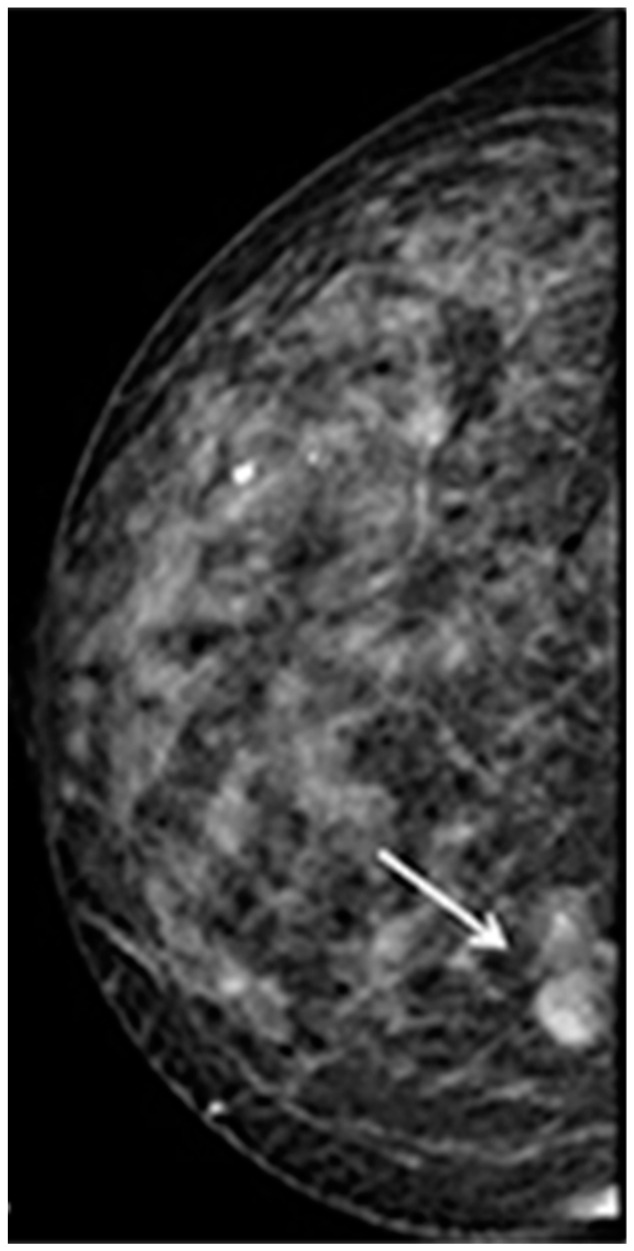
Digital cranio-caudal mammographic view: the lesion appears as a round opacity with regular edges located in the lower inner quadrant of the right breast (arrow).

**Figure 3:**
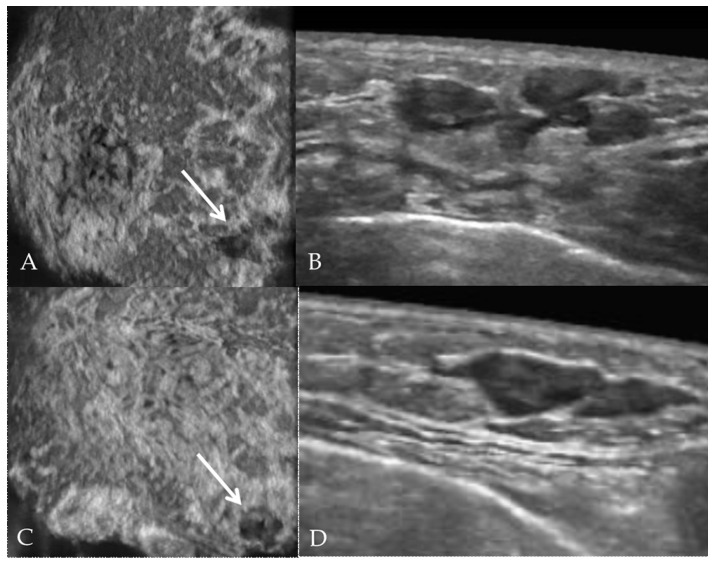
Automated breast ultrasound scan on coronal (**A**,**C**) and axial planes (**B**,**D**). The lesion appears as an oval hypoechoic nodule with regular edges, mimicking duct ectasia (arrows).

The patient reported that, immediately after the diagnosis of SS, she received methotrexate and prednisone for a few months; currently, she still is only undergoing antihypertensive and hydroxychloroquine therapy and shows no relevant signs of SS (e.g., parotid enlargement or eye/mouth dryness). Routine laboratory tests were within normal limits. As to the breast lesion, a painless swelling of small size was detected on palpation; conventional mammography showed a small radiolucency with regular and well-defined margins of the lower inner quadrant (Figure 2), while ultrasound examination highlighted a round opacity with regular edges (Figure 3A–D).

Regardless of the benign appearance on both imaging investigations, a US-guided core needle biopsy of the lesion was performed; unexpectedly, the subsequent histopathological examination showed diffuse proliferation of small to medium-sized lymphoid cells, with slightly hyperchromatic nuclei, without plasmacytic differentiation, accompanied by stromal sclerosis and residual atrophic ducts.

**Figure 4:**
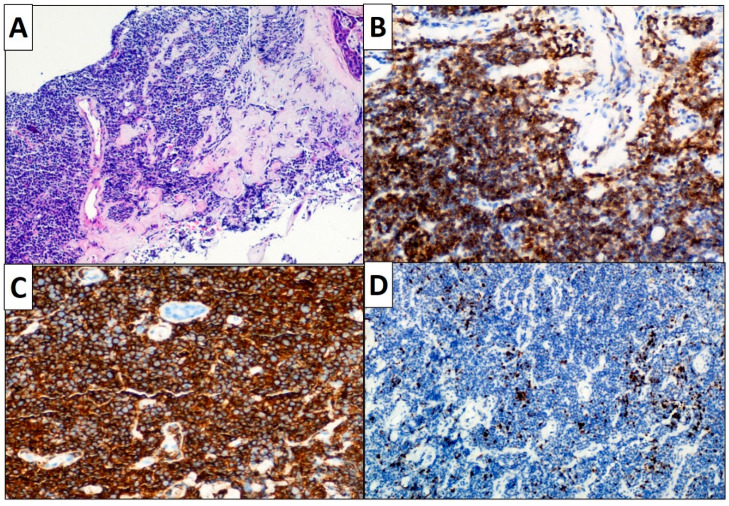
Primary breast marginal zone NHL is characterized by diffuse proliferation of small to medium-sized lymphoid cells and accompanied by stromal sclerosis and residual atrophic ducts ((**A**): hematoxylin and eosin, original magnification 40×). The neoplastic lymphocytes are strongly immunoreactive for bcl2 ((**B**): original magnification 200×) and CD20 ((**C**): original magnification 200×). The immunohistochemical stain for Ki67 shows a very low proliferative index, pointing at an “indolent” lymphoma ((**D**): original magnification 100×).

Complimentary immunohistochemical investigations were performed to confirm the purportedly monoclonal nature of the lymphoid proliferation, highlighting the vast majority of infiltrating lymphocytes being of the B phenotype, and distinctly immunoreactive for CD20, CD79a and bcl2, while no immunoreactivity for CD3, CD5, CD10, CD23, cyclin D1, bcl6 and LEF1 was detected in lymphoid neoplastic cells. Less than 10% tumor cells displayed nuclear anti-Ki 67 (MIB 1) positivity. MALT gene rearrangement, involving the MALT1 locus at chromosome 18q21, using a MALT FISH Split Signal DNA Probe, could not be demonstrated. All such findings pointed at the diagnosis of primary extranodal MZL of MALT (MALT lymphoma) (Figure 4A–D). No lymphadenopathy, spleen enlargement, bone marrow involvement or other localizations of the disease were detected. Peripheral blood tests revealed the persistence of anti-SS A and B (anti-SSA/SSB) antibodies, cryoglobulins and low levels of C4 and C3.

## 3. Discussion

Primary breast lymphomas (PBL) represent approximately 1% of all NHLs, 1.7–2.2% of all extra-nodal NHLs and 0.04–0.5% of all malignancies of the breast. [18,19,20,21] Around 9% of all primary breast lymphomas are MZLs of MALT and usually manifest an indolent clinical behavior [21,22,23].

It is generally accepted that chronic inflammatory diseases (such as SS, Hashimoto thyroiditis, *Borrelia Burgdorferi* dermatitis, Helicobacter pylori-associated chronic gastritis and HCV, HHV8, EBV and HTLV-1 infections) may play a role in lymphoma development, resulting in the transition from polyclonal B cell activation into monoclonal expansion of B-lymphocytes. The transition into B-cell NHL only affects a minority of the aforementioned patients harboring chronic inflammatory diseases and has been associated with increased overall disease mortality rate [5,6,7,8,9,11,24].

Bizjak et al. in 2015 [25] extensively reviewed the role of inflammation related to breast silicone implants and other silicone prostheses (such as cardiac pacemakers and defibrillators, cardiac valvular and testicular/penile prostheses) in lymphoma development. They assumed that chronic inflammation in predisposed individuals could evolve into severe scarring of peri-implant tissues and to persistent activation of the local/systemic immune system. Such a pathogenetic mechanism was demonstrated for a distinct type of NHL, namely breast implant-associated anaplastic large T-cell lymphoma (BI-ALCL).

Patients with autoimmune diseases represent 5% of NHL patients, and NHL surely is the most severe complication occurring during SS patients’ follow-up; [5,6,7,8,9,10,24] nevertheless, the pathogenetic mechanism for such association has not been clarified [1,2,3,4,5,6,9,10,11,24,26].

As widely discussed by Vasaitis et al. in a recent population-based study [26], data available in the literature on NHL in SS patients are not at all uniform, and even basic data on gender preferences and overall lymphoma prevalence are not well established yet [26]. In view of the mostly indolent clinical behavior of NHL in SS patients, and in consideration of a median follow-up time that rarely exceeds 10 years in most reported studies, an accurate definition of epidemiologic data, including prevalence, can be hardly assessed. In addition, while several studies have reported on the occurrence of NHL in the breast, these were not focused on possible associations between breast NHL and SS [15,26,27,28,29].

In a recent update on prognostic markers of lymphoma development in SS patients, Retamozo et al. (2019) [30] stated that such patients show a seven-fold increased risk of lymphoma in comparison with systemic lupus erythematosus patients, four-fold with rheumatoid arthritis patients and globally >10-fold in comparison with the general population [5,30].

The same authors listed, point-to-point evaluated and discussed the different prognostic/predictive factors outlined in previously published studies, such as epidemiologic markers (age and sex), clinical markers (parotid enlargement, dry mouth and eyes, arthralgias, splenomegaly, lymphadenopathy, skin purpura/vasculitis), laboratory markers (systemic activity, hypergamma/raised IgG, CD4/CD8 ≤ 0.8, raised beta2-microglobulin, raised B-cell activating factors, anemia, leukopenia, lymphopenia, neutropenia, ANA, rheumatoid factor, Anti-Ro/La, low C4, C3 and CH 50 levels, cryoglobulins, mIgs) and histologic markers (focus score and ectopic primary or secondary follicle). They concluded that, although the association of more risk factors surely increases the risk of NHL, such prediction still remains imperfect; therefore, SS patients surely deserve closer follow-up, with attentive evaluation of the aforementioned risk factors, including cryoglobulin-related markers and increased EULAR SS disease activity index (ESSDAI), to more accurately identify patients at higher risk for SS-associated NHL [30,31].

As to primary breast lymphomas (PBLs), they are usually detected as palpable masses, associated or not with axillary lymph node enlargement, thus mimicking breast carcinoma or other breast neoplasms [32]. Furthermore, notwithstanding several attempts, no specific clinical or imaging patterns have been reported for breast lymphomas [33,34,35,36]. Radiologically, as for the case reported herein, PBL more commonly resembles inflammatory lesions, such as lymphocytic mastitis [37], IgG4-related sclerosing mastitis [38] and cutaneous lymphoid hyperplasia [39].

Consequently, the diagnosis of breast MZL of MALT usually is based on morphologic examinations. At this regard, fine needle aspiration cytology, a minimally invasive procedure, was proven effective to accurately diagnose the most common non-neoplastic (e.g., fibrocystic disease) and neoplastic (e.g., fibroadenoma and carcinoma) breast lesions at a pre-operative stage; nevertheless, such a diagnostic procedure may be of limited value when dealing with lymphoid proliferations, which may not show unequivocal morphologic features or may require extensive immunohistochemical investigations to achieve the final diagnosis. Consequently, histological preparations are more frequently adopted in such cases, which may allow proper morphologic evaluation of the lymphoid populations, appropriate immunohistochemical characterization, along with the possible detection of genetic alterations by in situ hybridization techniques, whenever deemed necessary. In the current case, all necessary morphologic and ancillary procedures could be carried out, even if dealing with small tissue fragments, thus highlighting the appropriateness of core needle biopsy as a diagnostic tool for PBL.

Based on the data available in the literature about PBL-SS association [40,41,42,43,44], and the current theories about lymphoma prevalence at immune-privileged sites [45], we can assume that the diagnosis of SS-related lymphoid proliferations, especially when occurring in the breast, currently is very challenging and would probably benefit from wider studies including SS patients with prolonged follow-up (>10 years). Therefore, we may suggest more attentive monitoring for lymphoma development in those SS patients who display higher risk factors (such as palpable purpura, low C4, mixed monoclonal cryoglobulinemia) and to incorporate breast surveillance in such patients.
